# Ultra-Wideband Temperature Dependent Dielectric Spectroscopy of Porcine Tissue and Blood in the Microwave Frequency Range

**DOI:** 10.3390/s19071707

**Published:** 2019-04-10

**Authors:** Sebastian Ley, Susanne Schilling, Ondrej Fiser, Jan Vrba, Jürgen Sachs, Marko Helbig

**Affiliations:** 1 Biosignal Processing Group, Technische Universität Ilmenau, 98693 Ilmenau, Germany; susanne.schilling@tu-ilmenau.de (S.S.); marko.helbig@tu-ilmenau.de (M.H.); 2 Department of Biomedical Technology, Faculty of Biomedical Engineering, CTU in Prague, 272 01 Kladno, Czech Republic; ondrej.fiser@fbmi.cvut.cz; 3 Department of Electromagnetic Field, Faculty of Electrical Engineering, CTU in Prague, 160 00 Prague, Czech Republic; vrba@fel.cvut.cz; 4 Electronic Measurements and Signal Processing Group, Technische Universität Ilmenau, 98693 Ilmenau, Germany; juergen.sachs@tu-ilmenau.de; 5 ILMSENS GmbH, 98693 Ilmenau, Germany

**Keywords:** dielectric spectroscopy, temperature dependent dielectric properties of blood, fat, liver, muscle, M-sequence, ultra-wideband, open-ended coaxial probe

## Abstract

The knowledge of frequency and temperature dependent dielectric properties of tissue is essential to develop ultra-wideband diagnostic technologies, such as a non-invasive temperature monitoring system during hyperthermia treatment. To this end, we characterized the dielectric properties of animal liver, muscle, fat and blood in the microwave frequency range from 0.5 GHz to 7 GHz and in the temperature range between 30 °C and 50 °C. The measured data were modeled to a two-pole Cole-Cole model and a second-order polynomial was introduced to fit the Cole-Cole parameters as a function of temperature. The parametric model provides access to the dielectric properties of tissue at any frequency and temperature in the specified range.

## 1. Introduction

Electromagnetic waves in the microwave frequency range offer a possibility to examine a non-transparent medium under test (MUT), because they are capable of penetrating a variety of materials, including biological tissues, without ionization. The dielectric properties determine how electromagnetic waves propagate within the MUT. The knowledge of these properties is essential for a wide variety of medical applications such as microwave breast cancer imaging [1,2,3,4,5,6] or microwave ablation and temperature monitoring [7,8,9,10,11]. The latter scenario is a promising approach to monitor the temperature distribution inside of the body during thermal therapies (e.g., hyperthermia), which support oncological treatments (e.g., chemotherapy or radiotherapy). During hyperthermia treatment the temperature is increased by around 4–8 °C in the tumor region by means of radio frequencies, ultrasound or microwaves, whereby it is important that the temperature does not exceed the upper limit. Microwave temperature monitoring offers a non-invasive and painless approach compared to the commonly used invasive fiber optic catheters [12]. The approach of microwave temperature monitoring is based on the temperature dependent dielectric properties of tissue.

**Figure 1:**
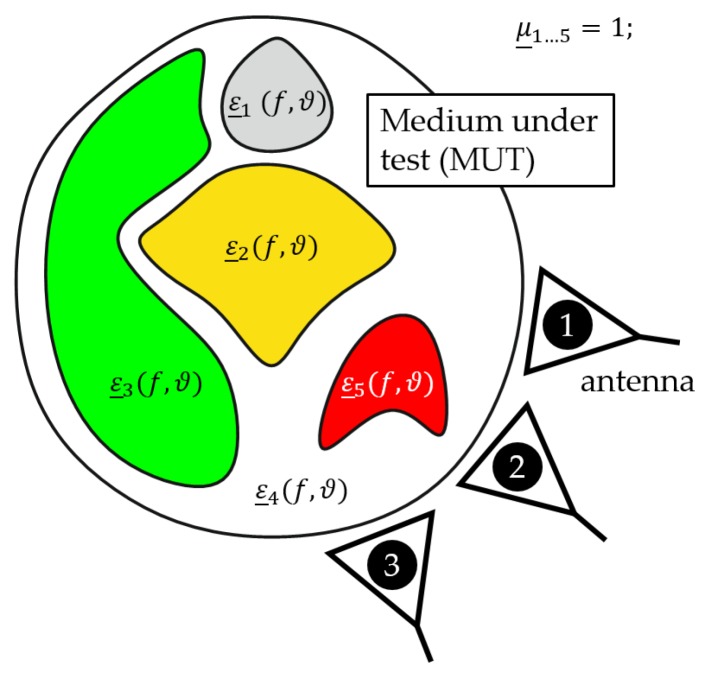
Generic temperature monitoring setup for medical applications.

Figure 1 illustrates a generic scenario for medical applications (e.g., neck, breast) where transmitting antennas emit low power electromagnetic waves into the MUT. The propagation of the electromagnetic waves (reflection, absorption, transmission and dispersion) inside of the MUT are determined by the tissue specific complex permittivity $\underline{\varepsilon }$ depending on the frequency *f* and temperature ϑ. If we consider such a scenario without any contrast agents (e.g., magnetic nanoparticles), the permeability can be set to $\underline{\mu }=1$ due to the non-magnetic behavior of tissue. If the temperature is increased in a specific region (e.g., tumor), the scattering of the electromagnetic waves will change due to the temperature dependency of the dielectric properties. The changes of the backscattered electromagnetic waves can be measured by means of ultra-wideband (UWB) technology [11,13]. To evaluate the differences in a measured UWB-signal corresponding to the temperature change, it is necessary to attain knowledge about the temperature dependent complex permittivity of the different kinds of tissue in the microwave frequency range.

A large number of studies have investigated the dielectric properties of various tissues over a wide frequency range [14,15,16,17,18], but most investigations only consider the complex permittivity at a constant temperature. Only a few studies have reported dielectric properties of tissue depending on temperature over a wide frequency range. Lazebnik et al. presented temperature dependent dielectric properties of porcine liver ranging from room temperature up to around 60 °C in the frequency range of 0.5 GHz to 20 GHz [19]. Further studies investigated the temperature dependency of blood in the frequency range lower than 1 GHz [20] and in the wide frequency range from 1 Hz to 40 GHz [21] as well as between 0.4 GHz and 20 GHz [22]. Furthermore, there are some more studies investigating the temperature dependency, but only for discrete frequencies. A literature survey of temperature dependent dielectric properties is presented by Rossmann and Haemmerich [23].

The purpose of this study is to determine the temperature dependent dielectric properties of tissue and blood. Measurements are performed by open-ended coaxial probe technique which is commonly used for dielectric spectroscopy of biological tissue [24]. We investigate liver, muscle and blood as high water content tissues and fat as a low water content tissue. Furthermore, the influence of the extraction time on the dielectric properties as well as the impact of heating and cooling measurements on the dielectric properties are observed. We derive temperature dependent dielectric spectroscopy data sets in the frequency range of 0.5 GHz to 7 GHz and in the temperature range between 30 °C and 50 °C. Finally, we introduce a two-pole Cole-Cole model for each tissue to describe the spectrum over the considered frequency range and we use a quadratic fit to model the temperature dependency of the Cole-Cole parameters.

## 2. Material and Methods

### 2.1. Measurement Setup

**Figure 2:**
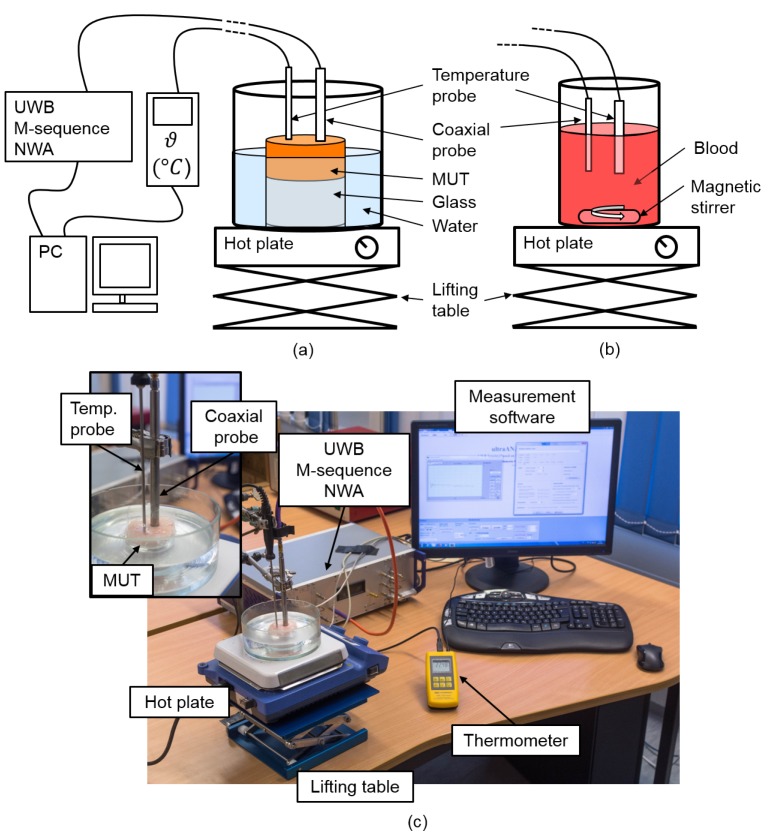
Measurement setup for temperature dependent ultra-wideband (UWB) spectroscopy of (**a**) tissue and (**b**) blood using an UWB M-sequence network analyzer (NWA) connected to a coaxial probe. A temperature probe connected to a high precision thermometer acquires the temperature. Both UWB radar signals and temperature are stored in parallel. (**c**) Laboratory measurement setup for tissue analysis.

The temperature dependent dielectric properties were measured using an UWB M-sequence network analyzer (NWA) based on a one port measurement (S11) [25]. Further details of the M-sequence technology can be found in Ref. [26]. Figure 2 shows the measurement setup for temperature dependent spectroscopy of tissue and blood which is mostly identical with the one presented in Ref. [27]. In both cases, tissue as well as blood analysis, the NWA was connected to the performance probe (N1501A performance probe, Keysight Technologies, Santa Clara, CA, USA). We acquired 189 equidistant data points in the frequency range from 0.5 GHz to 7 GHz corresponding to a frequency resolution of approximately 35 MHz. The temperature was acquired between 30 °C and 50 °C in steps of 1 °C by a temperature probe connected to a high precision thermometer (GMH 3750, GHM Messtechnik GmbH, Remscheid, Germany). In the case of tissue analysis, the samples had a cylindrical shape with a diameter of 6 cm and a height between 1 cm and 1.5 cm which is sufficient to avoid disturbing reflections (e.g., from the glass bottom) as proven by Hagl et al. [28] and Meaney et al. [29] as well as in our own experiments. The samples were wrapped in plastic film with two holes on the surface to ensure a direct contact of the measurement probes with the MUT. The samples were heated in a water bath as illustrated in Figure 2a. Concerning blood analysis, the probes were immersed a few millimeters into the blood which was heated by a hot plate as depicted in Figure 2b. A magnetic stirrer was used to avoid a sedimentation of the cellular components during the measurement.

### 2.2. Temperature Dependent Dielectric Properties

The temperature dependent dielectric properties are described by the complex relative permittivity

(1) $$\begin{array}{c} \underline{\varepsilon }\left(\omega ,\vartheta \right)=\varepsilon^{\prime }\left(\omega ,\vartheta \right)-i\varepsilon^{\prime \prime }\left(\omega ,\vartheta \right) \end{array}$$

and the effective conductivity

(2) $$\begin{array}{c} \sigma \left(\omega ,\vartheta \right)=\omega \varepsilon_{0}\varepsilon^{\prime \prime }\left(\omega ,\vartheta \right) \end{array}$$

where the real part $\varepsilon^{\prime }$ represents the relative permittivity, the imaginary part $\varepsilon^{\prime \prime }$ the relative dielectric loss, $\varepsilon_{0}$ the permittivity of free space, ω=2πf the angular frequency and ϑ the temperature.

### 2.3. Temperature Dependent Cole-Cole Model

In this section we determine a temperature and frequency dependent model to reduce the size of the measured data. Furthermore, such a model enables to calculate the relative permittivity and effective conductivity at any temperature and frequency in the specified range. The frequency dependent dielectric properties of tissue are mainly characterized by the three relaxation regions, α, β, and γ occurring in the low, medium and high frequency range [30]. Gabriel introduced a four pole Cole-Cole model to describe the spectrum over a frequency range from 10 Hz to 100 GHz [31]. In this study we consider the frequency range between 0.5 GHz and 7 GHz where the dielectric properties are dominated by the γ dispersion, but in tissue the dispersion regions frequently overlap [32]. For this reason we decided to use a two-pole Cole-Cole model, which includes the β and γ dispersion to fit the data in the frequency range corresponding to

(3) $$\begin{array}{c} \underline{\varepsilon }\left(\omega ,\vartheta \right)=\varepsilon_{\infty }\left(\vartheta \right)+\frac{\Delta \varepsilon_{1}\left(\vartheta \right)}{1+{\left(i\omega \tau_{1}\left(\vartheta \right)\right)}^{1-\alpha_{1}}}+\frac{\Delta \varepsilon_{2}\left(\vartheta \right)}{1+{\left(i\omega \tau_{2}\left(\vartheta \right)\right)}^{1-\alpha_{2}}}+\frac{\sigma_{s}\left(\vartheta \right)}{i\omega \varepsilon_{0}} \end{array}$$

where $\varepsilon_{\infty }$ represents the permittivity at very high frequencies, $\Delta \varepsilon_{1,2}$ are the dispersion amplitudes and $\tau_{1,2}$ the corresponding relaxation times and $\sigma_{s}$ the static conductivity. The parameters $\alpha_{1,2}$ are empirical distribution parameters describing the broadening of the dispersion.

In the first step we use the Levenberg-Marquart algorithm to fit the Cole-Cole parameters to the experimental data for each temperature corresponding to Equation (3). To limit the number of fitting parameters, $\alpha_{1}$ and $\alpha_{2}$ are set to a constant value for each tissue. The initial values for the calculation of $\varepsilon_{\infty ,fit},\Delta \varepsilon_{1,fit},\Delta \varepsilon_{2,fit},\tau_{1,fit}$, $\tau_{2,fit}$ and $\sigma_{s,fit}$ are set to the data presented by Gabriel [31]. First, we determine the Cole-Cole parameters $\varepsilon_{\infty ,fit^{*}},\Delta \varepsilon_{1,fit^{*}},\Delta \varepsilon_{2,fit^{*}},\tau_{1,fit^{*}}$ and $\tau_{2,fit^{*}}$ to fit the real part of the complex permittivity by minimizing the mean absolute error (difference between modeled and measured relative permittivity) across the specified frequency range. Based on this, we compute $\sigma_{s,fit^{*}}$ to finalize the fit of the imaginary part by minimizing the mean absolute difference between modeled and measured relative dielectric loss across all frequency points. The results of this first part (symbolized by the * in the above-mentioned Cole-Cole parameters) of the temperature dependent Cole-Cole fitting procedure are depicted in Figures 7, 10, 13, 16 by the blue circles.

In a second step, we use a second order polynomial to fit the temperature dependency of the Cole-Cole parameters corresponding to

(4) $$\begin{array}{cc} \varepsilon_{\infty ,fit}\left(\vartheta \right) & =A_{1}\vartheta^{2}+B_{1}\vartheta +C_{1}\\ \Delta \varepsilon_{1,fit}\left(\vartheta \right) & =A_{2}\vartheta^{2}+B_{2}\vartheta +C_{2}\\ \tau_{1,fit}\left(\vartheta \right) & =A_{3}\vartheta^{2}+B_{3}\vartheta +C_{3}\\ \Delta \varepsilon_{2,fit}\left(\vartheta \right) & =A_{4}\vartheta^{2}+B_{4}\vartheta +C_{4}\\ \tau_{2,fit}\left(\vartheta \right) & =A_{5}\vartheta^{2}+B_{5}\vartheta +C_{5}\\ \sigma_{s,fit}\left(\vartheta \right) & =A_{6}\vartheta^{2}+B_{6}\vartheta +C_{6} \end{array}$$

which is also suggested by Lazebnik [19]. The coefficients $A_{n},B_{n},C_{n}\left(n=1\ldots 6\right)$ for the different tissues are presented in the following sections.

Finally, the temperature dependent dielectric properties can be computed by

(5) $$\begin{array}{cc} {\underline{\varepsilon }}_{fit}\left(\omega ,\vartheta \right) & =\varepsilon_{fit}^{\prime }\left(\omega ,\vartheta \right)-i\varepsilon_{fit}^{\prime \prime }\left(\omega ,\vartheta \right)\\ & =\varepsilon_{\infty ,fit}\left(\vartheta \right)+\frac{\Delta \varepsilon_{1,fit}\left(\vartheta \right)}{1+{\left(i\omega \tau_{1,fit}\left(\vartheta \right)\right)}^{1-\alpha_{1}}}+\frac{\Delta \varepsilon_{2,fit}\left(\vartheta \right)}{1+{\left(i\omega \tau_{2,fit}\left(\vartheta \right)\right)}^{1-\alpha_{2}}}+\frac{\sigma_{s,fit}\left(\vartheta \right)}{i\omega \varepsilon_{0}} \end{array}$$

To quantify the quality of the fitting procedure we analyze the difference between the measured data and the results derived by the two-pole Cole-Cole model corresponding to Equation (5) by

(6) $$\begin{array}{cc} \delta \varepsilon_{measured,fit}^{\prime }\left(\omega ,\vartheta \right) & =\varepsilon_{measured}^{\prime }\left(\omega ,\vartheta \right)-\varepsilon_{fit}^{\prime }\left(\omega ,\vartheta \right)\\ \delta \sigma_{measured,fit}\left(\omega ,\vartheta \right) & =\sigma_{measured}\left(\omega ,\vartheta \right)-\sigma_{fit}\left(\omega ,\vartheta \right) \end{array}$$

with $\sigma_{fit}\left(\omega ,\vartheta \right)=\omega \varepsilon_{0}\varepsilon_{fit}^{\prime \prime }\left(\omega ,\vartheta \right)$.

## 3. Preliminary Investigations

The measurements presented in this section are intended to determine the working conditions for the temperature dependent spectroscopy of tissue and blood.

### 3.1. Influence of Storage Time

First, we observed the influence of the storage time between excision and measurement of porcine tissue and blood on the dielectric properties. Recent studies indicated that there is no significant difference in dielectric properties between in-vivo and ex-vivo investigations [17,19,33]. Lazebnik postulated that cell death has an insignificant influence on the dielectric properties at microwave frequencies. Furthermore, it is described that some fluid loss and changes in oxygen tension, pH and temperature will occur after excision, whereby only fluid loss and temperature are expected to affect the dielectric properties in the microwave frequency range [19]. Farrugia et al. also summarized that the changes between in in-vivo and ex-vivo dielectric properties are caused by tissue hydration [17]. The goal of this study is not to investigate the differences between in-vivo and ex-vivo measurements of tissue, but we want to clarify if the storage time (time between excision and measurement) has a significant influence on the dielectric properties. Therefore, we obtained porcine tissue and blood from local slaughter houses. The samples were stored in a fridge between excision and measurement. The dielectric properties were measured corresponding to the measurement setup presented above.

**Figure 3:**
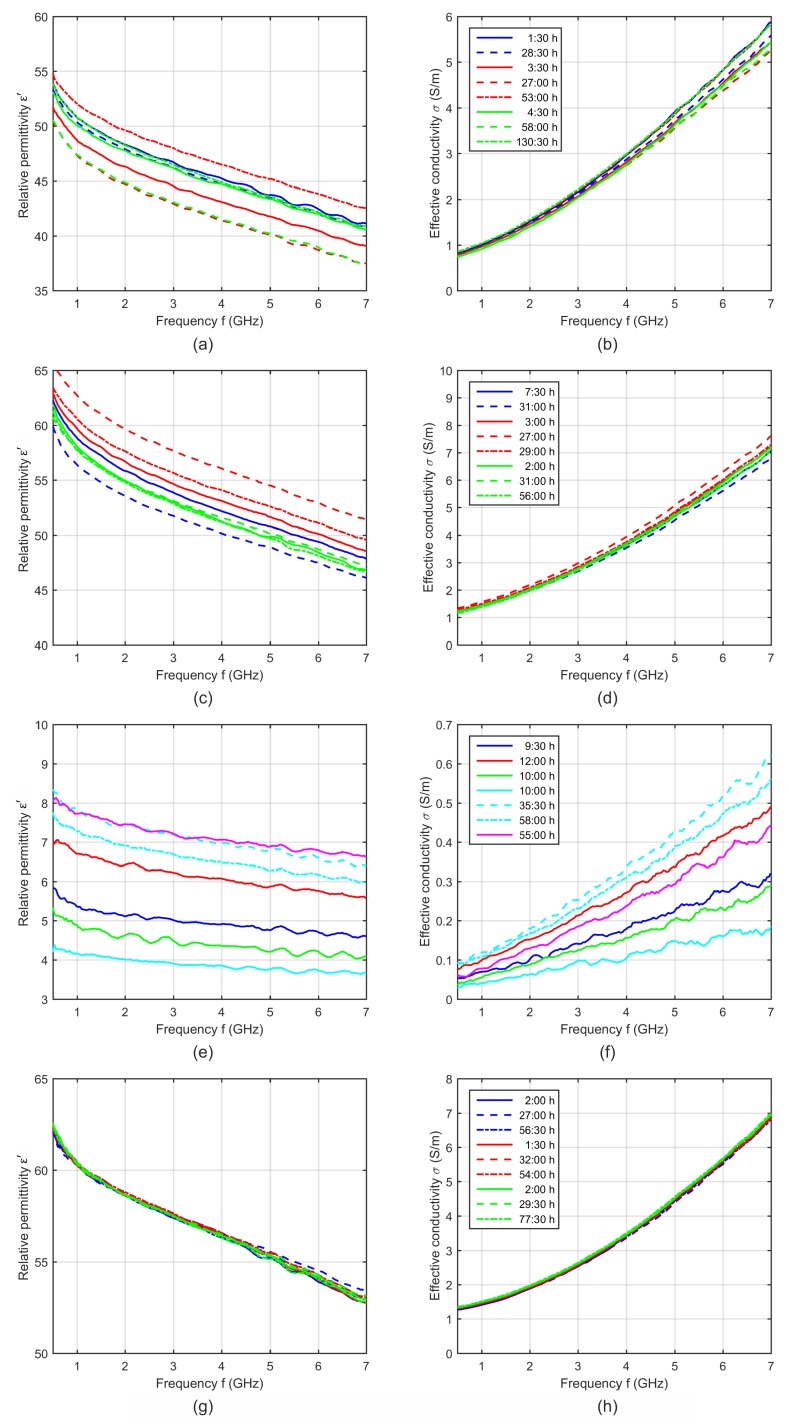
Relative permittivity and effective conductivity of (**a**,**b**) porcine liver, (**c**,**d**) muscle, (**e**,**f**) fat and (**g**,**h**) blood at 37 °C as a function of frequency for different storage times. Each curve shows the dielectric properties of one sample, whereby curves of the same color indicate samples of the same animal.

In general, we measured one and the same piece of tissue only one time in order to exclude effects due to denaturation. Therefore, we analyzed several tissue pieces from one animal spread over time from a few hours after extraction until several days after extraction. Figure 3 shows the relative permittivity and effective conductivity of porcine tissue and blood as a function of frequency for different storage times.

Assuming a significant influence of the storage time, a decreasing water content due to progressive dehydration would mainly influence the dielectric properties in the microwave frequency range. This effect would lead to continuously decreasing permittivity instead of the irregular behavior, as can be seen in Figure 3. For example, considering the results of muscle samples of different animals (Figure 3c,d), the sample with the longest storage time of the first animal (blue dashed curves) shows the lowest permittivity and conductivity, whereby both samples with the longer storage time of the second animal (red dashed curves) show the highest dielectric properties. Moreover, the measurements of one and the same animals (e.g., the animal represented by the red curves) illustrate significant differences between the samples without any correlation to the storage time, whereas other measurements (e.g., from the animal representing by the green curves) show nearly no differences. The same irregular behavior can be identified in the curves of liver and fat (Figure 3a,b,e,f).

Due to these findings, the storage time itself cannot be the origin of these differences. The intra-individual tissue differences (permittivity differences between various samples of one tissue type of one animal due to the tissue inhomogeneity) as well as the inter-individual differences (permittivity differences between samples of one tissue type of different animals) are much more dominant than the influence of the storage time. This knowledge and the result that all blood measurements were nearly identical (Figure 3g,h) allows us to average the measurements of one tissue type independently from the storage time.

### 3.2. Measurement Procedure of Temperature Dependency

**Figure 4:**
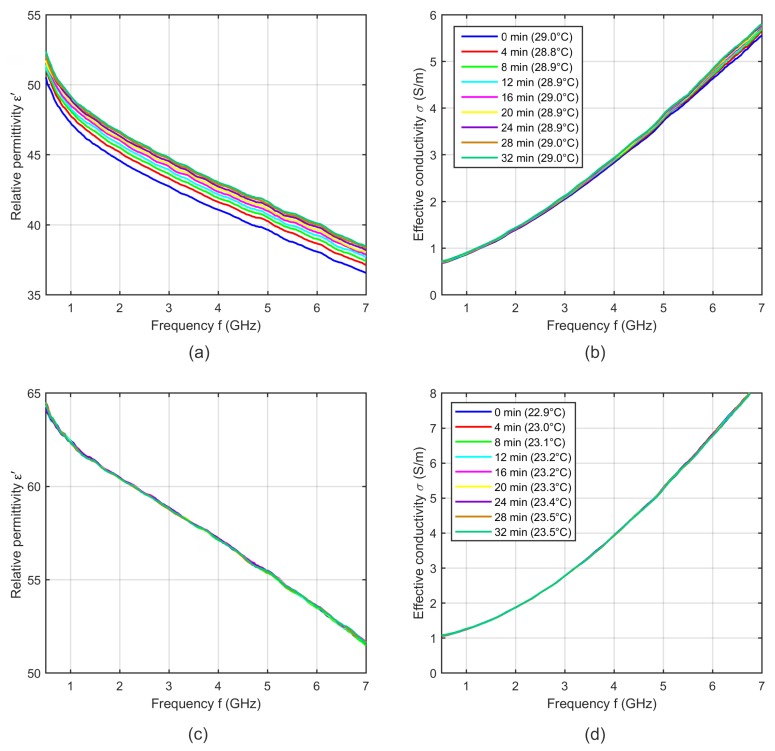
(**a**) Relative permittivity and (**b**) effective conductivity of one porcine liver sample as a function of frequency at a constant temperature over a time period of 32 min. (**c**) Relative permittivity and (**d**) effective conductivity of one porcine blood sample as a function of frequency at a constant temperature over a time period of 32 min. The blue curves indicate the measurement immediately after coaxial probe positioning.

In a first step, we investigated the dielectric properties at a constant temperature depending on the time after probe positioning. Figure 4a,b shows exemplarily the relative permittivtiy and effective conductivity of one liver sample over a time period of 32 min. The results show a non-linear time dependent increase of the dielectric properties after positioning the coaxial probe on the tissue sample. This effect also occurs in muscle and fat. It seems that the pressure of the coaxial probe on the tissue sample causes a change in the dielectric properties, whereby this effect decreases with an increasing waiting time. As a consequence we decided to wait 30 min after probe positioning before we started the temperature dependent dielectric spectroscopy of tissue. After the waiting period, the time dependent changes of the dielectric properties are significantly lower compared to the variations immediately after probe positioning as shown in Figure 4a,b. In principle, these remaining small changes should be minimized as much as possible, but it seems that they cannot be eliminated completely. Their extent to the error of the absolute permittivity value is nearly negligible but in comparison to the temperature dependent variations they can be significant. Thus, it is very important that they are temperature independent.

In the case of blood, the measurement procedure started directly after immersing the probe into the liquid, because the systematic error mentioned above does not occur during the measurement of blood as illustrated in Figure 4c,d.

**Figure 5:**
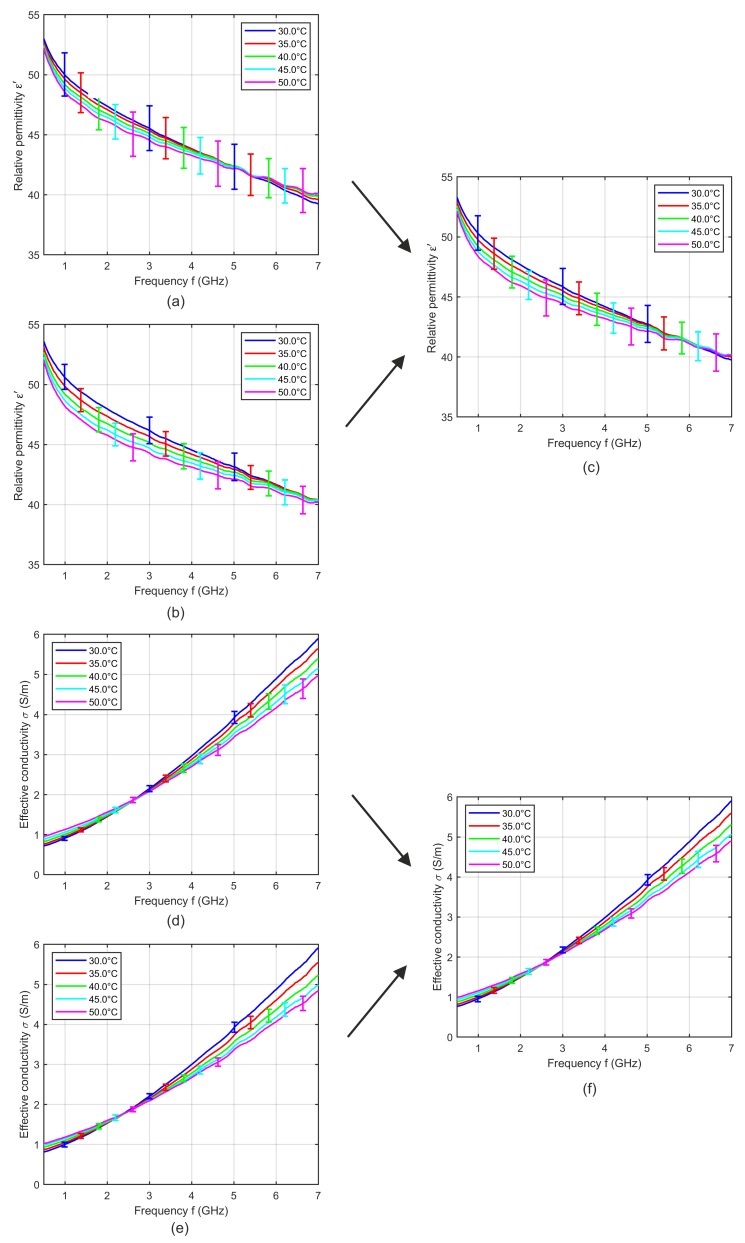
Mean relative permittivity and mean effective conductivity of six porcine liver samples acquired during (**a**,**d**) heating and (**b**,**e**) cooling cycle as a function of frequency at different temperatures. (**c**,**f**) Mean relative permittivity and mean effective conductivity of 12 porcine liver samples including six heating and six cooling measurements. Error bars indicate the corresponding standard deviation.

In a next step, we investigate the influence of measuring the dielectric properties during the heating and the cooling cycle. Figure 5a,d shows the relative permittivity and effective conductivity at five distinct temperatures averaged over six samples during the heating cycle and Figure 5b,e shows the temperature dependent dielectric properties averaged over six samples during the cooling cycle. The error bars indicate the corresponding standard deviation. The relative permittivity during heating cycle shows lower temperature dependency compared to the data recorded during cooling cycle. Furthermore, the intersection point of the relative permittivity occurred at around 5.2 GHz for measurements during heating, whereby the cross-over point shifts towards higher frequencies for the cooling cycle measurements. Lazebnik also described differences between heating and cooling cycles, whereby the results acquired during the heating cycle show repeatable anomalies [19], but the effects of the anomalies on the permittivity and conductivity are not described in detail. We suppose that the pressure dependent influence described in this section superimposes the temperature dependent changes of the dielectric properties of tissue. Due to the water content of tissue, we expect a decrease of the relative permittivity with increasing temperature. In the case of heating the temperature dependent decrease of the permittivity will be reduced by the time dependent increase of the permittivity (see Figure 5a). In contrast, the increasing permittivity during the cooling cycle measurement will be additionally raised by the time dependent permittivity increase (see Figure 5b). Therefore, we compute the averaged relative permittivity and effective conductivity over all samples including six measurements recorded during the heating and six measurements during the cooling cycle as shown in Figure 5c,f.

The averaging of identical numbers of heating and cooling measurements ensures that the low impact of the mentioned systematic error still remaining after 30 min waiting after probe positioning will be temperature independent. This is essential to obtain reliable temperature dependencies of tissues. We use this procedure to determine the temperature dependent dielectric properties of liver, muscle and fat.

**Figure 6:**
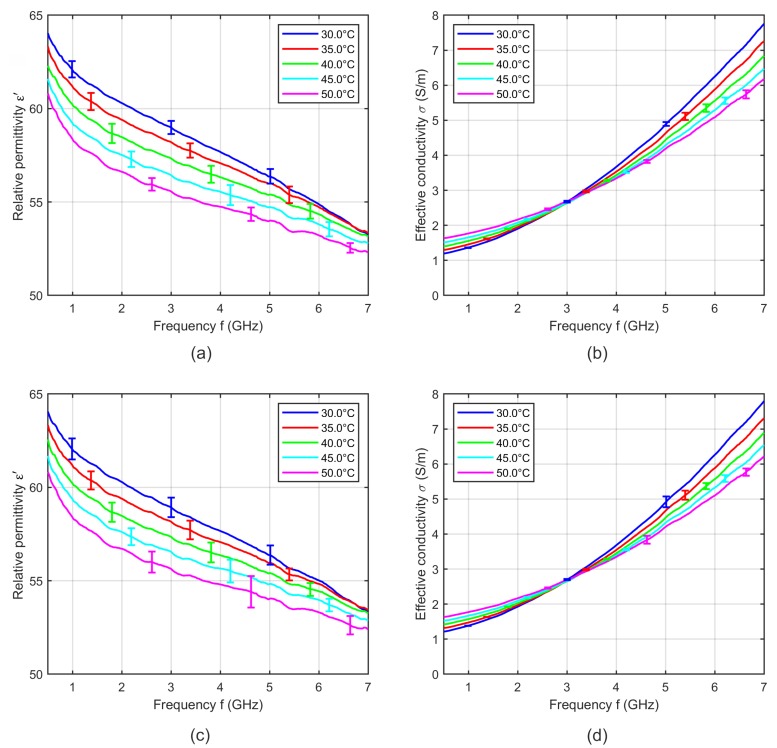
Mean relative permittivity and mean effective conductivity of three porcine blood samples acquired during (**a**,**b**) heating and (**c**,**d**) cooling cycle as a function of frequency at different temperatures. Error bars indicate the corresponding standard deviation.

Figure 6 shows the relative permittivity and effective conductivity of blood averaged over three samples recorded during heating and three samples during cooling with the corresponding standard deviation. In contrast to soft tissue, blood shows no differences between both cycles. Due to this finding, the dielectric properties of blood were recorded only during sample heating.

## 4. Results

In this section we present the temperature dependent properties of liver, muscle, fat and blood corresponding to the introduced temperature dependent two-pole Cole-Cole model. Due to the findings of the previous section, we applied the fitting procedure to the mean relative permittivity and mean effective conductivity over all samples for each tissue.

### 4.1. Liver

**Figure 7:**
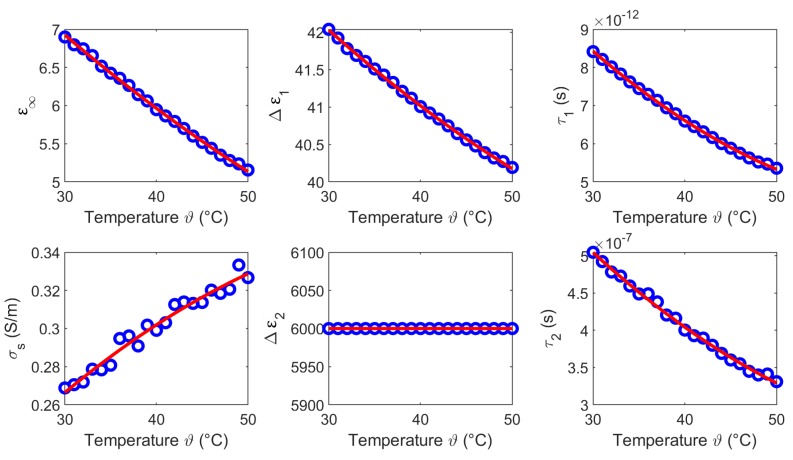
Temperature dependent Cole-Cole parameters (blue circles) of liver and the corresponding second order polynomial fit (red curves).

We derived the temperature dependent Cole-Cole parameters for each temperature based on the averaged measurements (see Figure 5c,f), whereby $\alpha_{1}$ and $\alpha_{2}$ are set to 0.2. Figure 7 shows the modeled temperature dependent Cole-Cole parameters (blue circles) as a function of temperature and the second order polynomial fits (red curves). The coefficients of the quadratic fits are summarized in Table 1.

**Figure 8:**
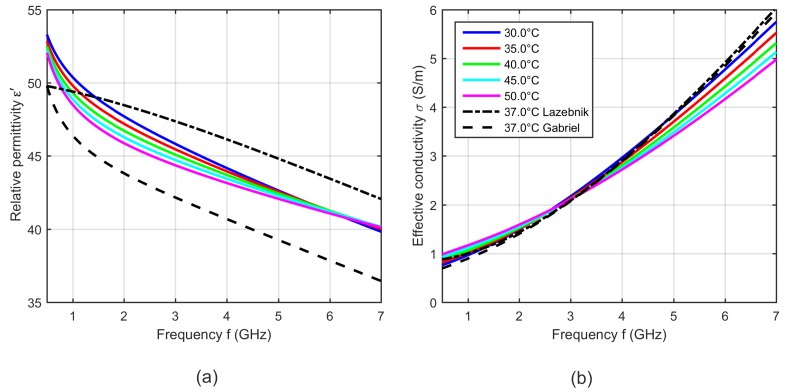
(**a**) Relative permittivity and (**b**) effective conductivity of porcine liver as a function of frequency at five different temperatures determined by the two-pole Cole-Cole model based on the derived temperature dependent Cole-Cole parameters. Black curves show the dielectric properties of liver reported by Lazebnik (bovine) [19] and Gabriel (ovine) [31] at 37 °C.

Figure 8 shows exemplarily five curves of the relative permittivity and effective conductivity determined by the two-pole Cole-Cole model (see Equation (5)) with the temperature dependent Cole-Cole parameters computed by Equation (4) and the temperature coefficients summarized in Table 1.

The results shown in Figure 8 are within the range of the relative permittivity and effective conductivity at 37 °C presented by Lazebnik [19] and Gabriel [31]. The modeled temperature dependent dielectric properties show cross-over points, where the relative permittivity and the effective conductivity do not change with temperature. The relative permittivity has an intersection point at 6 GHz. Below this point, the relative permittivity decreases with increasing temperature and above the cross-over point this dependency reverses. The effective conductivity also shows one intersection point in the observed frequency range at 3 GHz, whereby the conductivity rises up with increasing temperature below the cross-over point. This dependency reverses for frequencies higher than 3 GHz. The observed trends are consistent with the results presented by Lazebnik [19].

**Figure 9:**
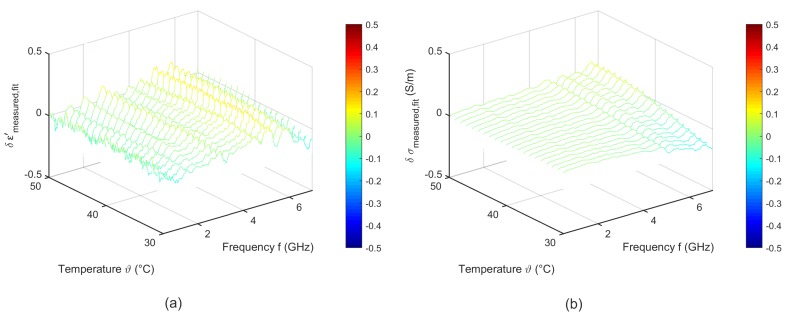
Difference between the measured data and the temperature dependent two pole Cole-Cole model of (**a**) relative permittivity and (**b**) effective conductivity of porcine liver.

To quantify the quality of the fitting procedure, Figure 9 shows the difference between the measured data and the results derived by the two-pole Cole-Cole model corresponding to Equation (6). The fitted curves agree very well with the experimental data. The deviations of the relative permittivity are lower than 0.2. The variations concerning the effective conductivity show slight differences to the measured data in the high frequency range for temperatures between 30 °C and 35 °C.

### 4.2. Muscle

**Figure 10:**
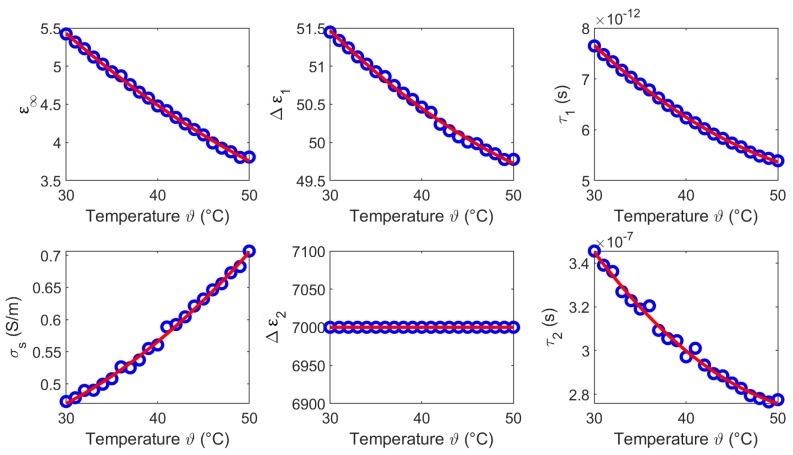
Temperature dependent Cole-Cole parameters (blue circles) of muscle and the corresponding second order polynomial fit (red curves).

The temperature dependent Cole-Cole parameters corresponding to the measured data are shown in Figure 10, whereby $\alpha_{1}$ and $\alpha_{2}$ are set to 0.18. The circles indicate the Cole-Cole coefficients of the two-pole Cole-Cole equation and the solid curves show the corresponding quadratic fits. The coefficients of the quadratic fits are summarized in Table 2.

**Figure 11:**
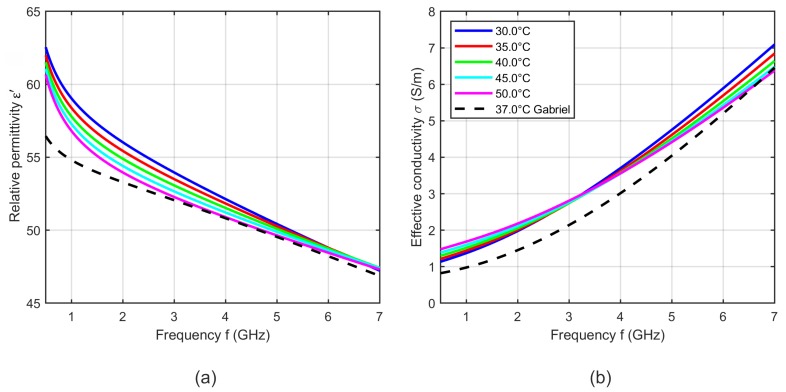
(**a**) Relative permittivity and (**b**) effective conductivity of porcine muscle as a function of frequency at different temperatures. Black curves show the dielectric properties of muscle reported by Gabriel [31] at 37 °C.

Figure 11 shows exemplarily five curves of the relative permittivity and effective conductivity determined by the two-pole Cole-Cole model (see Equation (5)) with the temperature dependent Cole-Cole parameters computed by Equation (4) and the temperature coefficients summarized in Table 2. The results are in agreement with the relative permittivity and effective conductivity at 37 °C presented by Gabriel [31]. Furthermore, the temperature dependent dielectric properties show similarities compared with the results of liver. Both the relative permittivity as well as the effective conductivity have a cross-over point in the considered frequency range. The relative permittivity decreases with increasing temperature below the intersection point at 6.5 GHz. The effective conductivity rises up with increasing temperature below the intersection point at 3 GHz. The trend reverses in both cases for frequencies above the corresponding cross-over point.

**Figure 12:**
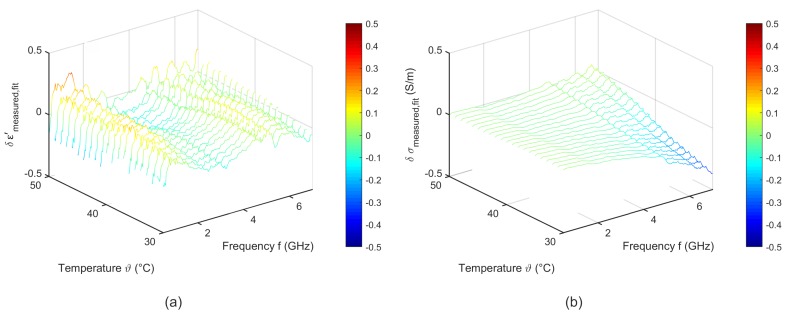
Difference between the measured and the temperature dependent two pole Cole-Cole model of (**a**) relative permittivity and (**b**) effective conductivity of porcine muscle.

Figure 12 shows the difference between the measured and the fitted data corresponding to Equation (6). The fitted curves show a good accordance to the experimental data. The deviations of the relative permittivity are lower than 0.3. The results of the fitting procedure concerning the effective conductivity show slight differences to the measured data in the high frequency range for temperatures between 30 °C and 35 °C.

### 4.3. Fat

**Figure 13:**
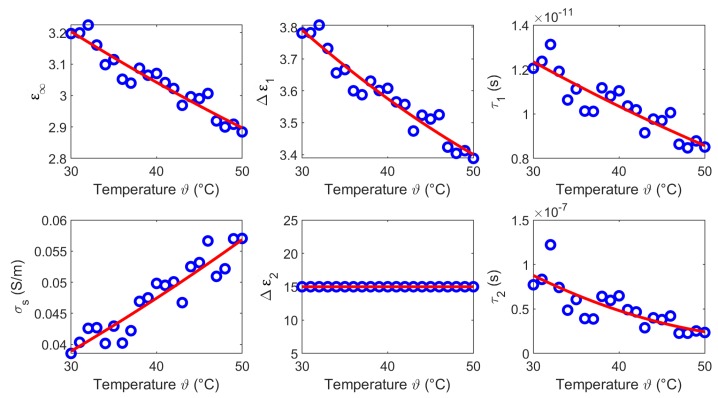
Temperature dependent Cole-Cole parameters (blue circles) of fat and the corresponding second order polynomial fit (red curves).

Figure 13 shows the temperature dependent Cole-Cole parameters corresponding to the experimental data, whereby $\alpha_{1}$ and $\alpha_{2}$ are set to 0.4. The circles show the modeled Cole-Cole parameters as a function of temperature and the solid curves illustrate the second order polynomial fit. The coefficients of the quadratic fits are summarized in Table 3.

**Figure 14:**
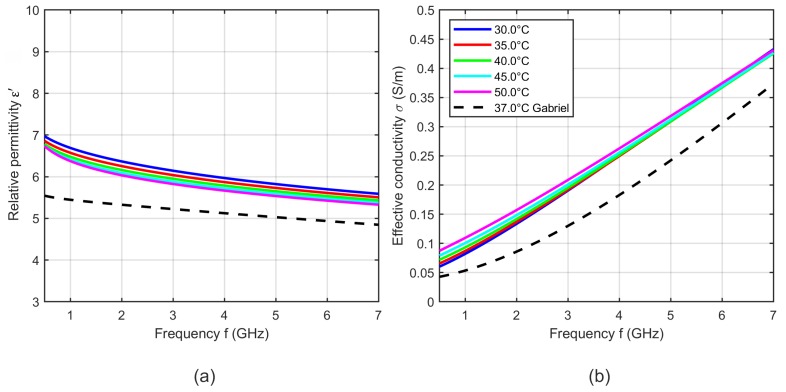
(**a**) Relative permittivity and (**b**) effective conductivity of porcine fat as a function of frequency at different temperatures. Black curves show the dielectric properties of fat reported by Gabriel [31] at 37 °C.

Figure 14 shows exemplarily five curves of the relative permittivity and effective conductivity determined by the two-pole Cole-Cole model (see Equation (5)) with the temperature dependent Cole-Cole parameters computed by Equation (4) and the temperature coefficients summarized in Table 3. The relative permittivity is nearly constant over the considered frequency range which agrees well with the data reported by Gabriel [31]. Furthermore, the permittivity decreases slightly with increasing temperature. The effective conductivity rises up with increasing temperature at low frequencies and shows almost no temperature dependency in the higher frequency range.

**Figure 15:**
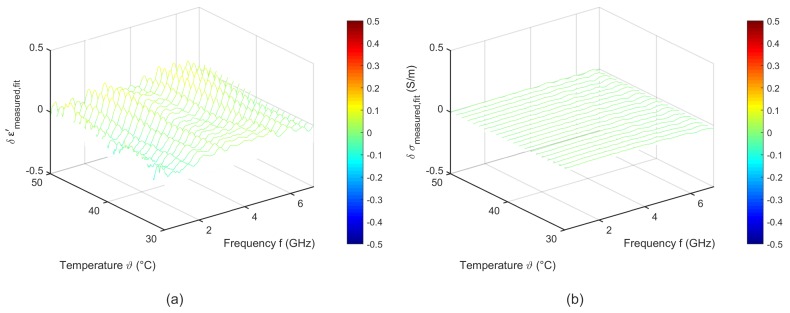
Difference between the measured data and the temperature dependent two pole Cole-Cole model of (**a**) relative permittivity and (**b**) effective conductivity of porcine fat.

Figure 15 shows the difference between the measured data and the fit corresponding to Equation (6). The deviations between fitted and experimental data are lower than 0.1 for the relative permittivity and less than 0.05 for the effective conductivity.

### 4.4. Blood

**Figure 16:**
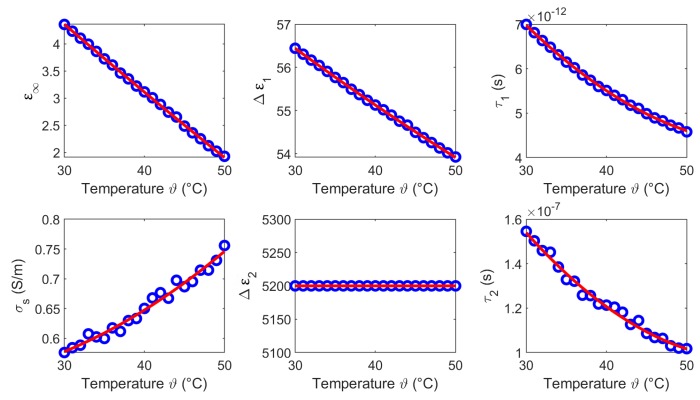
Temperature dependent Cole-Cole parameters (blue circles) of blood and the corresponding second order polynomial fit (red curves).

The temperature dependent Cole-Cole parameters based on the experimental data are depicted in Figure 16, whereby $\alpha_{1}$ and $\alpha_{2}$ are set to 0.1. The circles present the Cole-Cole coefficients of the two-pole Cole-Cole function and the corresponding second order polynomial fit. The coefficients of the quadratic fits are summarized in Table 4.

**Figure 17:**
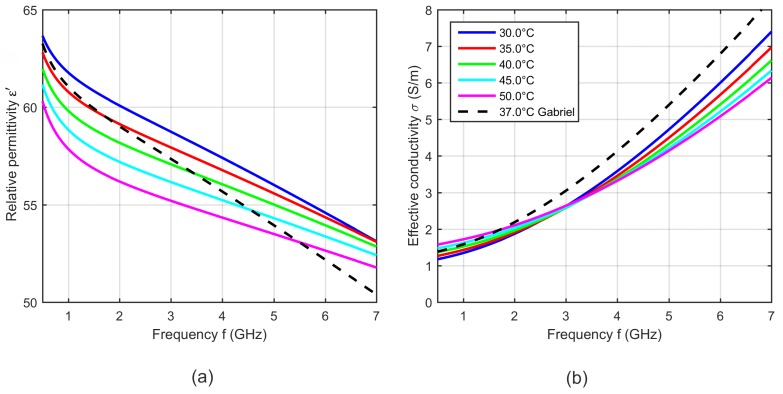
(**a**) Relative permittivity and (**b**) effective conductivity of porcine blood as a function of frequency at different temperatures. Black curves show the dielectric properties of blood reported by Gabriel [31] at 37 °C.

Figure 17 shows five curves of the relative permittivity and effective conductivity determined by the two-pole Cole-Cole model (see Equation (5)) with the temperature dependent Cole-Cole parameters computed by Equation (4) and the temperature coefficients summarized in Table 4. The curves show a slight deviation in slope compared to the data presented by Gabriel [31], but the dielectric properties are within the same range. The relative permittivity decreases with increasing temperature, whereby it is conspicuous that the temperature dependency is non-linear at high frequencies. This behavior is comparable to the temperature dependent dielectric properties of water [34,35]. The effective conductivity increases with rising temperature below the cross-over point at 3 GHz. This trend reverses at frequencies above the intersection point and is consistent with the results presented by Wolf et al. [21].

**Figure 18:**
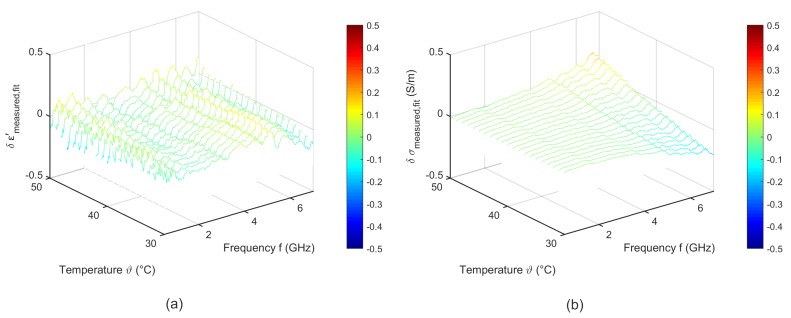
Difference between the measured data and the temperature dependent two pole Cole-Cole model of (**a**) relative permittivity and (**b**) effective conductivity of porcine blood.

Figure 18 shows the difference between the measured data and the fit corresponding to Equation (6). The fitted data agree with the measured ones. The deviations of the relative permittivity are lower than 0.2. The variations concerning the effective conductivity show slight differences to the measured data in the high frequency range.

### 4.5. Discussion

**Figure 19:**
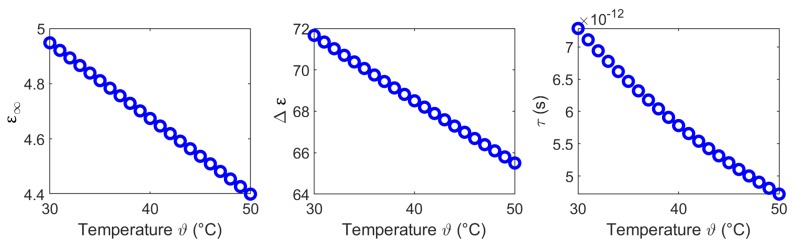
Temperature dependent model parameters of pure water corresponding to Kaatze [34].

The results of this section show that the dielectric properties as well as the temperature dependency are correlated to the water content of the investigated tissues. This is consistent with the fact that the dielectric properties in the microwave frequency range are dominated by the relaxation of water. The temperature dependent dielectric properties of pure water has been widely investigated by Kaatze [34] and Ellison [35]. The relative permittivity and the effective conductivity of pure water can be modeled by a one-pole model, because only one relaxation mechanism occurs in the microwave frequency range. Figure 19 illustrates the temperature dependent parameters of pure water corresponding to the model introduced by Kaatze [34]. The parameters $\varepsilon_{\infty },\Delta \varepsilon ,\tau$ decrease with increasing temperature similar to the Cole-Cole parameters ($\varepsilon_{\infty },\Delta \varepsilon_{1},\tau_{1}$) of the investigated tissues derived in this study. The static conductivity $\sigma_{s}$ rises with increasing temperature in all examined MUT. This trend concurs with the results for liver reported by Lazebnik et al. [19]. The parameters $\Delta \varepsilon_{2}$ and $\tau_{2}$ of the second Cole-Cole pole mainly influence the relative permittivity in the lower frequency range between 0.5 GHz and ≈2 GHz. This is indicated by the increasing relative permittivity in this region (see Figure 8, Figure 11, Figure 14 and Figure 17) which is not shown by the data of the one-pole Cole-Cole model of Lazebnik (see Figure 8a). The temperature dependence of the second pole is represented by the relaxation time $\tau_{2}$ of the respective tissues, whereby the dispersion amplitudes $\Delta \varepsilon_{2}$ are constant over the temperature as shown in Figure 7, Figure 10, Figure 13 and Figure 16.

Due to the high water content of liver and muscle between 73% and 78%, the permittivity and the conductivity are much higher compared to fat which has a water content between 5% and 20% [36]. Furthermore, the results of liver and muscle show nearly identical temperature dependent differences of their dielectric properties, whereby fat shows a low temperature dependent change of the relative permittivity and the effective conductivity. Blood has the highest temperature dependency of all investigated MUT, whereby the general behavior of the dielectric properties show similarities compared with liver and muscle, respectively. Furthermore, the effective conductivity of these three MUT has an intersection point at 3 GHz.

## 5. Conclusions

In this paper we presented a reliable measurement procedure to derive temperature dependent dielectric properties of tissue and blood. We measured the temperature dependent dielectric properties of ex-vivo porcine liver, muscle, fat and blood in the temperature range between 30 °C and 50 °C from 0.5 GHz up to 7 GHz. Furthermore, we presented a two pole Cole-Cole model combined with a second order polynomial fit to describe the frequency and temperature dependency of the dielectric properties in the specified ranges. The results of the fitting procedure agree well with the acquired data indicated by the low differences between the modeled and measured dielectric properties.

Our investigations show that the dielectric properties of each type of tissue (porcine liver, muscle, fat) show intra-individual as well as inter-individual differences. In comparison, porcine blood shows significant lower permittivity differences between various samples of one animal as well as between samples of different animals. Regardless of these differences, the measurements of all samples separately show a clear temperature dependence. Due to an efficient measurement procedure minimizing systematic errors, these dependencies could be reliably determined.

Concerning the targeted measurement scenario of temperature monitoring during hyperthermia treatment, the intra- and inter-individual variations will not be a problem because we want to detect the changes of dielectric properties by means of UWB radar differential imaging [37]. The knowledge of the temperature dependent dielectric properties of different types of tissue derived in this study are the fundamental base for modeling of antenna measurements in the future as well as for the development of appropriate signal processing algorithms interpreting the received UWB radar signals. Due to the similarities between human and porcine tissue, the obtained models can be used for numerical simulations of wave propagation inside of the human body.

On the one hand, the presented results illustrate the challenge of the measurement task of non-invasive temperature monitoring inside the human body and on the other hand its feasibility with our measuring UWB technology [37]. These findings are in accordance with our [11,13] and other [10] previous publications. Considering the frequency axis, it is obvious that the strongest temperature induced changes of the relative permittivity occur between 1 and 4 GHz. In cases of liver, muscle and blood and in contrast to the real part of the complex permittivity, the effective conductivity curves even show an intersection point around 3 GHz, indicating that the imaginary part of the complex permittivity does not generate temperature induced contrast at this frequency. These findings are important concerning the definition of optimal working conditions (e.g., the frequency band) and have to be considered in the development of a future UWB device for non-invasive temperature estimation.

## Figures and Tables

**Table 1:** Temperature coefficients of the second order polynomial fit to the temperature dependent Cole-Cole parameters of liver.

	*n*	$A_{n}$		$B_{n}$		$C_{n}$	
$\varepsilon_{\infty ,fit}$	1	0.6634 $\cdot 10^{-3}$	(K${}^{-2}$)	−0.1427	(K${}^{-1}$)	10.61	
$\Delta \varepsilon_{1,fit}$	2	0.8595 $\cdot 10^{-3}$	(K${}^{-2}$)	−0.1616	(K${}^{-1}$)	46.10	
$\tau_{1,fit}$	3	2.693	(fs · K${}^{-2}$)	−0.3705	(ps · K${}^{-1}$)	17.12	(ps)
$\Delta \varepsilon_{2,fit}$	4	0	(K${}^{-2}$)	0	(K${}^{-1}$)	6000	
$\tau_{2,fit}$	5	0.1259	(ns · K${}^{-2}$)	−18.83	(ns · K${}^{-1}$)	955.8	(ns)
$\sigma_{s,fit}$	6	−0.0436	(mS · K${}^{-2}$)	6.603	(mS · K${}^{-1}$)	107.6	(mS)

**Table 2:** Temperature coefficients of the second order polynomial fit to the temperature dependent Cole-Cole parameters of muscle.

	*n*	$A_{n}$		$B_{n}$		$C_{n}$	
$\varepsilon_{\infty ,fit}$	1	1.047 $\cdot 10^{-3}$	(K${}^{-2}$)	−0.1675	(K${}^{-1}$)	9.513	
$\Delta \varepsilon_{1,fit}$	2	1.495$\cdot 10^{-3}$	(K${}^{-2}$)	−0.2068	(K${}^{-1}$)	56.33	
$\tau_{1,fit}$	3	2.651	(fs · K${}^{-2}$)	−0.3266	(ps · K${}^{-1}$)	15.07	(ps)
$\Delta \varepsilon_{2,fit}$	4	0	(K${}^{-2}$)	0	(K${}^{-1}$)	7000	
$\tau_{2,fit}$	5	0.1053	(ns · K${}^{-2}$)	−11.89	(ns · K${}^{-1}$)	606.8	(ns)
$\sigma_{s,fit}$	6	0.2140	(mS · K${}^{-2}$)	−5.352	(mS · K${}^{-1}$)	437.5	(mS)

**Table 3:** Temperature coefficients of the second order polynomial fit to the temperature dependent Cole-Cole parameters of fat.

	*n*	$A_{n}$		$B_{n}$		$C_{n}$	
$\varepsilon_{\infty ,fit}$	1	0.0845 $\cdot 10^{-3}$	(K${}^{-2}$)	−0.0221	(K${}^{-1}$)	3.789	
$\Delta \varepsilon_{1,fit}$	2	0.2014 $\cdot 10^{-3}$	(K${}^{-2}$)	−0.0356	(K${}^{-1}$)	4.679	
$\tau_{1,fit}$	3	1.163	(fs · K${}^{-2}$)	−0.2815	(ps · K${}^{-1}$)	19.74	(ps)
$\Delta \varepsilon_{2,fit}$	4	0	(K${}^{-2}$)	−0.0002	(K${}^{-1}$)	15.00	
$\tau_{2,fit}$	5	0.0779	(ns · K${}^{-2}$)	−9.398	(ns · K${}^{-1}$)	299.5	(ns)
$\sigma_{s,fit}$	6	0.0043	(mS · K${}^{-2}$)	0.5504	(mS · K${}^{-1}$)	18.50	(mS)

**Table 4:** Temperature coefficients of the second order polynomial fit to the temperature dependent Cole-Cole parameters of blood.

	*n*	$A_{n}$		$B_{n}$		$C_{n}$	
$\varepsilon_{\infty ,fit}$	1	0.2158 $\cdot 10^{-3}$	(K${}^{-2}$)	−0.1399	(K${}^{-1}$)	8.362	
$\Delta \varepsilon_{1,fit}$	2	0.4829 $\cdot 10^{-3}$	(K${}^{-2}$)	−0.1652	(K${}^{-1}$)	60.96	
$\tau_{1,fit}$	3	2.865	(fs · K${}^{-2}$)	−0.3479	(ps · K${}^{-1}$)	14.84	(ps)
$\Delta \varepsilon_{2,fit}$	4	0	(K${}^{-2}$)	0	(K${}^{-1}$)	5200	
$\tau_{2,fit}$	5	0.0712	(ns · K${}^{-2}$)	−8.314	(ns · K${}^{-1}$)	339.2	(ns)
$\sigma_{s,fit}$	6	0.1434	(mS · K${}^{-2}$)	−3.063	(mS · K${}^{-1}$)	540.6	(mS)

## References

[1] Fear E.C., Bourqui J., Curtis C., Mew D., Docktor B., Romano C. (2013). Microwave breast imaging with a monostatic radar-based system: A study of application to patients. IEEE Trans. Microw. Theory Tech..

[2] Scapaticci R., Bellizzi G., Catapano I., Crocco L., Bucci O.M. (2014). An effective procedure for MNP-enhanced breast cancer microwave imaging. IEEE Trans. Biomed. Eng..

[3] Preece A.W., Craddock I., Shere M., Jones L., Winton H.L. (2016). MARIA M4: Clinical evaluation of a prototype ultrawideband radar scanner for breast cancer detection. J. Med. Imaging.

[4] Wörtge D., Moll J., Krozer V., Bazrafshan B., Hübner F., Park C., Vogl T. (2018). Comparison of X-ray-Mammography and Planar UWB Microwave Imaging of the Breast: First Results from a Patient Study. Diagnostics.

[5] O’Loughlin D., O’Halloran M., Moloney B.M., Glavin M., Jones E., Elahi M.A. (2018). Microwave breast imaging: Clinical advances and remaining challenges. IEEE Trans. Biomed. Eng..

[6] Helbig M., Dahlke K., Hilger I., Kmec M., Sachs J. (2012). Design and Test of an Imaging System for UWB Breast Cancer Detection. Frequenz.

[7] Scapaticci R., Bellizzi G.G., Cavagnaro M., Lopresto V., Crocco L. (2017). Exploiting Microwave Imaging Methods for Real-Time Monitoring of Thermal Ablation. Int. J. Antennas Propag..

[8] Kidera S., Neira L.M., Van Veen B.D., Hagness S.C. (2018). TDOA-based microwave imaging algorithm for real-time microwave ablation monitoring. Int. J. Microw. Wirel. Technol..

[9] Meaney P.M., Zhou T., Fanning M.W., Geimer S.D., Paulsen K.D. (2008). Microwave thermal imaging of scanned focused ultrasound heating: Phantom results. Int. J. Hyperth..

[10] Haynes M., Stang J., Moghaddam M. (2014). Real-time microwave imaging of differential temperature for thermal therapy monitoring. IEEE Trans. Biomed. Eng..

[11] Fiser O., Helbig M., Sachs J., Ley S., Merunka I., Vrba J. (2018). Microwave Non-invasive Temperature Monitoring Using UWB Radar for Cancer Treatment by Hyperthermia. Prog. Electromagn. Res..

[12] Schena E., Tosi D., Saccomandi P., Lewis E., Kim T. (2016). Fiber optic sensors for temperature monitoring during thermal treatments: An overview. Sensors.

[13] Ley S., Fiser O., Merunka I., Vrba J., Sachs J., Helbig M. Preliminary Investigations for Non-invasive Temperature Change Detection in Thermotherapy by Means of UWB Microwave Radar. Proceedings of the 2018 40th Annual International Conference of the IEEE Engineering in Medicine and Biology Society (EMBC).

[14] Gabriel S., Lau R.W., Gabriel C. (1996). The dielectric properties of biological tissues: II. Measurements in the frequency range 10 Hz to 20 GHz. Phys. Med. Biol. Phys. Med. Biol..

[15] Lazebnik M., Popovic D., McCartney L., Watkins C.B., Lindstrom M.J., Harter J., Sewall S., Ogilvie T., Magliocco A., Breslin T.M. (2007). A large-scale study of the ultrawideband microwave dielectric properties of normal, benign and malignant breast tissues obtained from cancer surgeries. Phys. Med. Biol..

[16] Fornes-Leal A., Garcia-Pardo C., Frasson M., Pons Beltrán V., Cardona N. (2016). Dielectric characterization of healthy and malignant colon tissues in the 0.5-18 GHz frequency band. Phys. Med. Biol..

[17] Farrugia L., Wismayer P.S., Mangion L.Z., Sammut C.V. (2016). Accurate in vivo dielectric properties of liver from 500 MHz to 40 GHz and their correlation to ex vivo measurements. Electromagn. Biol. Med..

[18] Porter E., Salahuddin S., La Gioia A., Elahi M.A., Shahzad A., Kumar A., Kilroy D., O’Halloran M. (2018). Characterization of the Dielectric Properties of the Bladder Over the Microw. Range. IEEE J. Electromagn. RF Microw. Med. Biol..

[19] Lazebnik M., Converse M.C., Booske J.H., Hagness S.C. (2006). Ultrawideband temperature-dependent dielectric properties of animal liver tissue in the microwave frequency range. Phys. Med. Biol..

[20] Jaspard F., Nadi M. (2002). Dielectric properties of blood: An investigation of temperature dependence. Physiol. Meas..

[21] Wolf M., Gulich R., Lunkenheimer P., Loidl A. (2011). Broadband dielectric spectroscopy on human blood. Biochim. Biophys. Acta Gen. Subj..

[22] Salahuddin S., O’Halloran M., Porter E., Farrugia L., Bonello J., Sammut C.V., Wismayer P.S. (2017). Effects of standard coagulant agents on the dielectric properties of fresh human blood. IEEE Trans. Dielectr. Electr. Insul..

[23] Rossmann C., Haemmerich D. (2014). Review of Temperature Dependence of Thermal Properties, Dielectric Properties, and Perfusion of Biological Tissues at Hyperthermic and Ablation Temperatures. Crit. Rev. Biomed. Eng..

[24] La Gioia A., Porter E., Merunka I., Shahzad A., Salahuddin S., Jones M., O’Halloran M. (2018). Open-Ended Coaxial Probe Technique for Dielectric Measurement of Biological Tissues: Challenges and Common Practices. Diagnostics.

[25] Hilger I., Dahlke K., Rimkus G., Geyer C., Seifert F., Kosch O., Thiel F., Hein M., Scotto F., Schwarz U. (2013). ultraMEDIS—Ultra-Wideband Sensing in Medicine. Ultra-Wideband Radio Technologies for Communications, Localization and Sensor Applications.

[26] Sachs J. (2012). Handbook of Ultra-Wideband Short-Range Sensing.

[27] Ley S., Fiser O., Merunka I., Vrba J., Sachs J., Helbig M. Preliminary Investigations for Reliable Temperature Dependent UWB Dielectric Spectroscopy of Tissues and Tissue Mimicking Phantom Materials. Proceedings of the 12th European Conference on Antennas and Propagation (EuCAP 2018).

[28] Hagl D.M., Popovic D., Hagness S.C., Booske J.H., Okoniewski M. (2003). Sensing volume of open-ended coaxial probes for dielectric characterization of breast tissue at microwave frequencies. IEEE Trans. Microw. Theory Tech..

[29] Meaney P.M., Gregory A.P., Seppala J., Lahtinen T. (2016). Open-Ended Coaxial Dielectric Probe Effective Penetration Depth Determination. IEEE Trans. Microw. Theory Tech..

[30] Gabriel C., Gabriel S., Corthout E. (1996). The dielectric properties of biological tissues: I. Literature survey. Phys. Med. Biol..

[31] Gabriel S., Lau R.W., Gabriel C. (1996). Physics in Medicine & Biology. The dielectric properties of biological tissues: III. Parametric models for the dielectric spectrum of tissues. Phys. Med. Biol. Phys. Med. Biol..

[32] Foster K.R., Schwan P.S. (1996). Dielectric Properties of Tissues. Handbook of Biological Effects of Electromagnetic Fields.

[33] Peyman A., Holden S., Gabriel C. (2005). Dielectric Properties of Tissues at Microwave Frequencies. Mobile Telecommunications and Health Research Programme Final Report.

[34] Kaatze U. (1989). Complex permittivity of water as a function of frequency and temperature. J. Chem. Eng. Data.

[35] Ellison W.J. (2007). Permittivity of pure water, at standard atmospheric pressure, over the frequency range 0–25 THz and the temperature range 0–100 °C. J. Phys. Chem. Ref. Data.

[36] Pethig R., Kell D.B. (1987). The passive electrical properties of biological systems: Their significance in physiology. Phys. Med. Biol..

[37] Sachs J., Ley S., Just T., Chamaani S., Helbig M. (2018). Differential ultra-wideband microwave imaging: Principle application challenges. Sensors.

